# Guillain-Barré syndrome in times of pandemics

**DOI:** 10.1136/jnnp-2020-324230

**Published:** 2020-07-30

**Authors:** Sonja E Leonhard, David R Cornblath, Hubert P Endtz, James J Sejvar, Bart C Jacobs

**Affiliations:** 1 Neurology, Erasmus MC, Rotterdam, The Netherlands; 2 Neurology, Johns Hopkins University, Baltimore, Maryland, USA; 3 Medical Microbiology & Infectious Diseases, Erasmus MC, Rotterdam, The Netherlands; 4 Fondation Mérieux, Lyon, France; 5 National Center for Emerging and Zoonotic Infectious Diseases, Centers for Disease Control and Prevention, Atlanta, Georgia, USA; 6 Neurology and Immunology, Erasmus MC, Rotterdam, The Netherlands

**Keywords:** guillain-barre syndrome, virology, neurovirology

## The SARS-CoV-2 pandemic is the last in line of several epidemics of infectious diseases that have been linked to the Guillain-Barré syndrome (GBS). As threats of epidemics of emerging infectious diseases persist, this is the time to learn from the past and to advance our response to future outbreaks in terms of research and management of GBS.

In the past decade, the world confronted several pandemics of emerging infectious diseases including Zika virus and most recently Severe Acute Respiratory Syndrome Coronavirus 2 (SARS-CoV-2). One of the neurological complications reported in relation to these infectious diseases is the Guillain-Barré syndrome (GBS), a rapidly progressive immune-mediated polyradiculoneuropathy that can cause paresis in all limbs, cranial and respiratory muscles.^1–3^ Approximately 20% require admission at an intensive care unit (ICU), and 2%–12% die, depending on the care available.^4^

In the past, research responses investigating a possible link between GBS and outbreaks of infectious diseases or vaccines have been delayed. This is problematic as healthcare institutions need to be able to prepare for increased incidences in patients with GBS, and public health personnel need to identify any possible mitigating factors. History now seems to repeat itself when case reports of SARS-CoV-2-related GBS are mounting, and disquiet over a possible association increases. As threats of epidemics of emerging infectious diseases persist, this is the time to learn from the past and to advance our response to future outbreaks in terms of research and management of GBS.

## Challenges and prospects in research preparedness

The first aims when studying a possible link between an infectious agent and GBS are to determine if a true association exists and to determine the impact in terms of frequency and severity. During an outbreak, observational cohorts are set up rapidly by clinicians, some of whom may lack experience in diagnosing and managing GBS due to the need to quickly mobilise personnel. These studies are often done at a single centre and not harmonised with GBS research from other centres, which can result in missing out of important clinical information.

How can one ensure a high-quality study within the limited time frame afforded by an infectious disease epidemic? Many hurdles must be overcome before recruitment can be started, and accurate and sufficient data collection is complex. Here, we list the most important hurdles and provide suggestions on how to deal with them.

### Study design: surveillance and case–control studies

To determine the impact in terms of frequency, a reliable and international surveillance platform for GBS incidence during and between epidemics, either active or passive, should be in place to define the background incidence and to detect an increase in cases. A surveillance system for acute flaccid paralysis (AFP) in children under the age of 15 was set up to eradicate polio and is operative globally (http://polioeradication.org/). The international community may benefit from introducing AFP surveillance for all ages or for GBS specifically.

To determine an association between GBS and an infectious agent, a cohort study with a case–control design is necessary. A predefined research protocol should be developed that is feasible in different healthcare infrastructures and easy to activate and use, to ensure a high-quality study within a limited time frame. Critical requirements for the study include clear case definitions for GBS and the collection of data on the clinical and electrophysiological phenotype, as this can be associated with a specific infectious agent and may provide evidence of an association. To study the impact for patients, outcome of at least 6–12 months with validated outcome measures should be recorded.

Such a protocol would be supported by a network of neurologists, such as the Inflammatory Neuropathy Consortium of the Peripheral Nerve Society, and can be based on the protocol of the International GBS Outcome Study, that is running in 19 countries and is also used by other research groups.^5 6^ Existing networks such as The Global Health Network could help to make the existence of such a protocol widely known.^7^ Inspiration can be drawn from large international research consortia on infectious diseases, such as the International Severe Acute Respiratory and emerging Infection Consortium (ISARIC) and the Platform for European Preparedness Against (Re-)emerging Epidemics (PREPARE) that assure and prepare an agile research response to outbreak-prone infectious diseases (https://www.prepare-europe.eu/; https://isaric.tghn.org/).

### Funding application and ethical permission

**Figure 1:**
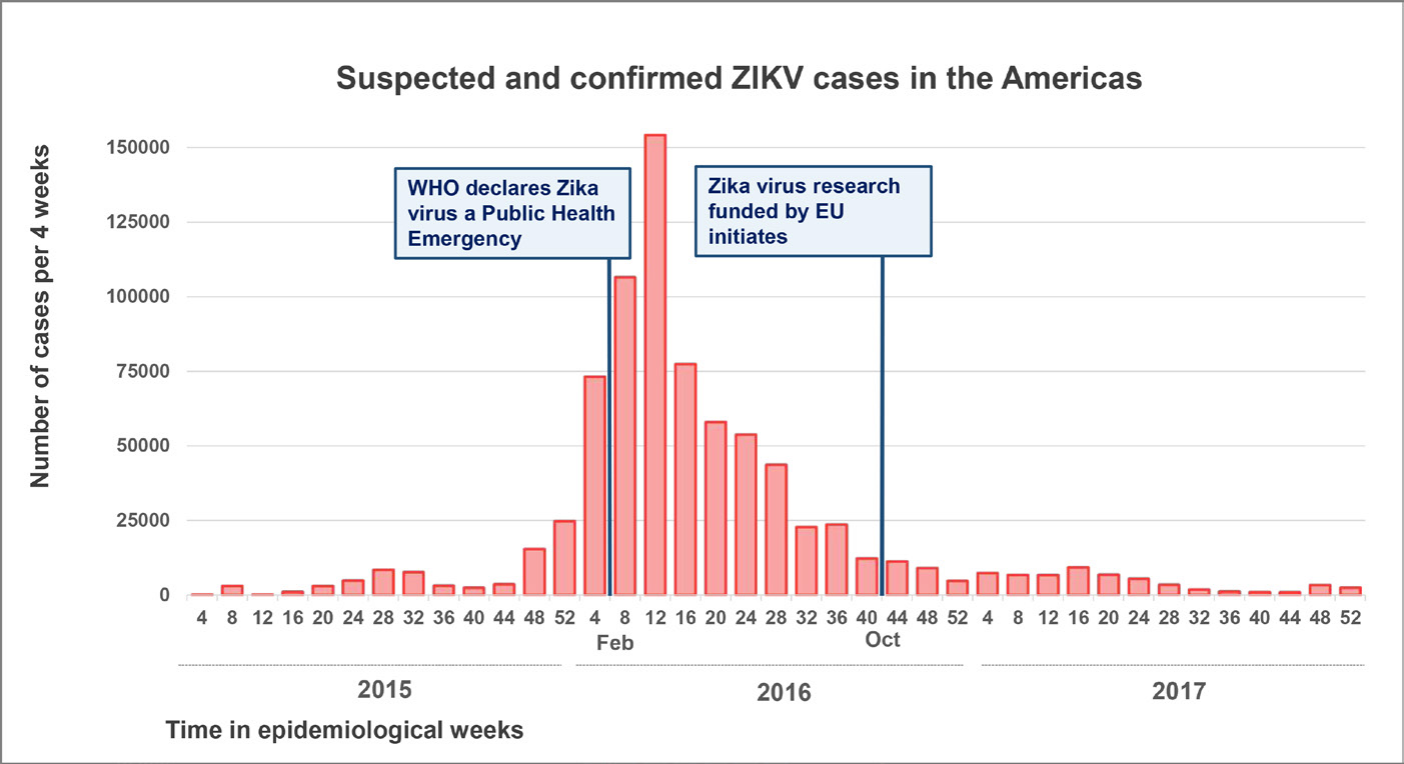
Suspected and confirmed ZIKV cases reported in the Americas by the WHO between January 2015 and December 2017, displayed per four epidemiological weeks.^25^ In October 2016, the ZikaPLAN consortium was able to initiate with funding from the EU as part of the Horizon 2020 programme.^10^ EU, European Union.

The time between application and receipt of funding and between submission and acceptance by an ethical review board is usually several months.^8 9^ This sequential process therefore often leads to significant delays. A recent example is the Zika virus pandemic that peaked at the beginning of 2016 when WHO declared it a Public Health Emergency of International Concern. By the time the Zika virus research consortia could initiate their work with funding from the European Union in October 2016, the peak of the epidemic had passed, and most participating researchers still needed to go through ethical approval^10^ (figure 1).

Fortunately, there are already initiatives in place to accelerate the process of grant application and ethical review during an outbreak. The Global Research Collaboration for Infectious Disease Preparedness (GLOPID-R) joins together major public and private research funding organisations to facilitate the mobilisation of resources and the immediate start of critical research in an outbreak situation (https://www.glopid-r.org/).^11 12^ Legal, ethical, logistical and administrative barriers that delay a research response at the peak of a health crisis could be addressed by making funding available during interpandemic periods, which can be used to develop standardised study protocols and research networks, with an additional budget to support infrastructure when the next outbreak occurs.

The idea of a ‘central’ or ‘universal’ institutional review board (IRB) in which institutional review could be fast-tracked in situations of emergent infectious diseases has recently been launched by the National Institutes of Health. Furthermore, in some jurisdictions, events of public health emergency can bypass complete IRB approval, thus shortening the time to implementation.^13 14^

### Collecting and sharing data and biosamples

As GBS is a rare disease (1–2 per 100 000 per year), a multicentre or even multinational approach is generally necessary to capture a sufficient number of cases to provide evidence of an association and describe the clinical phenotype.^15^ Setting up a multicentre study is time-consuming, and increasingly complex privacy regulations further restrict the sharing of data and biosamples between institutions. Operational consortia allow for the continued multicentre collection of data and samples during an epidemic, although sharing of biosamples often still requires material transfer agreements. Having preapproved protocols and agreements ready for use upfront could accelerate this process.

## Challenges and opportunities in diagnosis and management

In case of a sudden increase in patients with GBS, clinicians with limited expertise in GBS may need to manage these patients, and availability of facilities and resources may run out. We expect limitations mainly in ICU beds and rehabilitation care, as this was also reported during the Zika virus outbreak in Brazil.^16^ These limitations are especially important in low-resource countries that often have suboptimal or malfunctional healthcare systems, a lack of health professionals and are hot spots for outbreaks of emerging infectious diseases.^17^ Here, we provide recommendations on how to safeguard good quality diagnosis and management of GBS during a pandemic.

### Guideline for management of GBS

Diagnosis, treatment and monitoring of GBS can be complicated as patients may present with non-specific symptoms and vary with respect to clinical severity, treatment response and outcome.^18^ Furthermore, there are several diseases that can be difficult to distinguish from GBS, such as critical illness neuropathy, which is now especially important as many patients are admitted to the ICU for extended periods of time due to coronavirus disease 2019 (COVID-19). Recently a 10-step evidence-based consensus guideline for GBS was developed in response to the Zika virus outbreak.^19^ This guideline was designed to be compact and easy-to-use and applicable in all healthcare settings. An online version of the guideline is supported by The Global Health Network (https://rede.tghn.org/gbs-flowchart-sample/introduction-gbs/). Its use may help improve the management of GBS during an outbreak.

### Availability of resources

The two proven effective therapeutics for GBS, intravenous immunoglobulin (IVIg) and plasmapheresis, are expensive, and unaffordable for many patients in low-resource countries. Furthermore, demand for IVIg has tripled in the past decades, and shortages may occur in times of crisis.^20 21^ New and affordable treatment options for GBS are therefore warranted. A pilot study on small volume plasma exchange showed potential, but the therapeutic efficacy needs to be determined.^22^

The COVID-19 pandemic has made it apparent that upscaling availability of ICU beds is necessary to prepare for future outbreaks of infectious diseases that cause acute respiratory distress. Prediction models for respiratory failure in patients with GBS, such as the Erasmus GBS Respiratory Insufficiency Score, may further relieve pressure from ICU facilities but need to be validated in non-Western countries.^23^ Now that more patients are recovering from COVID-19, lack of caretakers and beds in rehabilitation units is also increasingly becoming a problem.^24^ Upscaling availability is imperative to cope with this new wave of patients and will also be of use in a future outbreak of GBS.

## Conclusion

In the past decade, multiple pandemics of infectious diseases have been linked to increased incidence of GBS. Epidemics will continue to occur, and it is vital to advance preparedness in research and clinical management of GBS in an outbreak setting.
